# KL-Biome (Postbiotic Formulation of *Lactiplantibacillus plantarum* KM2) Improves Dexamethasone-Induced Muscle Atrophy in Mice

**DOI:** 10.3390/ijms25137499

**Published:** 2024-07-08

**Authors:** Yu-Jin Jeong, Jong-Hoon Kim, Ye-Jin Jung, Mi-Sun Kwak, Moon-Hee Sung, Jee-Young Imm

**Affiliations:** 1 Department of Foods and Nutrition, Kookmin University, Seoul 02707, Republic of Korea; jus3111@kookmin.ac.kr; 2 KookminBio Corporation, Seoul 02826, Republic of Korea; kbjh0701@kmbio.co.kr (J.-H.K.); jeongyj@kmbio.co.kr (Y.-J.J.); mskwak@kmbio.co.kr (M.-S.K.); smoonhee@kmbio.co.kr (M.-H.S.)

**Keywords:** sarcopenia, dexamethasone, postbiotics, KL-biome, gut microbiome

## Abstract

Sarcopenia refers to an age-related decrease in muscle mass and strength. The gut–muscle axis has been proposed as a promising target to alleviate muscle atrophy. The effect of KL-Biome—a postbiotic preparation comprising heat-killed *Lactiplantibacillus plantarum* KM-2, its metabolites, and an excipient (soybean powder)—on muscle atrophy was evaluated using dexamethasone (DEX)-induced atrophic C2C12 myoblasts and C57BL/6J mice. KL-Biome significantly downregulated the expression of genes (Atrogin-1 and MuRF1) associated with skeletal muscle degradation but increased the anabolic phosphorylation of FoxO3a, Akt, and mTOR in C2C12 cells. Oral administration of KL-Biome (900 mg/kg) for 8 weeks significantly improved muscle mass, muscle function, and serum lactate dehydrogenase levels in DEX-treated mice. KL-Biome administration increased gut microbiome diversity and reversed DEX-mediated gut microbiota alterations. Furthermore, it significantly increased the relative abundances of the genera *Subdologranulum*, *Alistipes*, and *Faecalibacterium prausnitzii*, which are substantially involved in short-chain fatty acid production. These findings suggest that KL-Biome exerts beneficial effects on muscle atrophy by regulating gut microbiota.

## 1. Introduction

Sarcopenia refers to an age-related reduction in skeletal muscle mass and function that adversely influences quality of life [1]. Although no standard diagnostic criteria exist, it reportedly affects at least 5–13% of individuals in the 60–70 year age group [2]. Muscle plays a significant role in glucose homeostasis and energy metabolism in the body, and decreased muscle mass raises the risk of chronic diseases such as osteoporosis, arthritis, obesity, and diabetes [3]. Muscle atrophy is a cellular mechanism caused by an imbalance between anabolic and catabolic pathways. In addition, increased inflammatory signals also lead to muscle wasting [4]. At a molecular level, the IGF-1/PI3K/AKT/mTOR pathway stimulates protein synthesis, while FoxO translocation to the nucleus triggers muscle breakdown via the unbiquitin-autophagy pathway [5].

The gut microbiota is considered a major contributor to host health and displays a close association with muscle mass. Germ-free mice exhibit decreased skeletal muscle mass, while gut microbiota transplantation results in a significant improvement in muscle atrophy. Moreover, the administration of short-chain fatty acids (**SCFAs**) has been found to partially counteract skeletal muscle damage in germ-free mice [6]. Gut–muscle crosstalk can be mediated by microbial metabolites, such as gut peptides, SCFAs, and cytokines [7,8]. This suggests that gut microbiota can improve sarcopenia by modulating chronic inflammation, energy metabolism, and insulin sensitivity [9].

*Lactiplantibacillus plantarum* is a commensal microorganism in the human gastrointestinal tract and is commonly found in various fermented foods [10]. Owing to its long history of safe use, *L. plantarum* has been classified as “Generally Regarded as Safe” by the Food and Drug Administration and is deemed suitable for the “Qualified Presumption of Safety” by the European Food Safety Authority [11]. *L. plantarum* has demonstrated diverse health benefits. The administration of *L. plantarum* C88 and *L. plantarum* 200655 reduced oxidative stress and promoted hepatic glutathione peroxidase activity in aged mice [12,13]. *L. plantarum* strains, such as *L. plantarum* TWK10 and *L. plantarum* HY7715, were found to improve muscle mass and function in a rodent model [14,15], with *L. plantarum* HY7715 postulated to potentially improve protein bioavailability; however, the detailed underlying mechanisms are yet to be elucidated.

Postbiotics are defined as non-viable microorganisms and/or their derivatives that provide health benefits to the host. They potentially provide superior therapeutic benefits to probiotics by conferring greater stability and safety [16]. KL-Biome is a formulated postbiotic ingredient comprising heat-killed *L. plantarum* KM-2, its metabolites, and an excipient. Soybean powder was used as the excipient to facilitate drying. This study aimed to evaluate the effects of KL-Biome on muscle atrophy using dexamethasone (DEX)-induced atrophic C2C12 myotubes and C57BL/6J mice. To achieve this goal, changes in muscle mass and function, serum biochemical markers, the expression of genes and proteins associated with skeletal muscle mass, and gut microbiota were systemically analyzed.

## 2. Results & Discussion

### 2.1. Effect of KL-Biome on Myotube Diameter in DEX-Induced Atrophic C2C12 Myotubes

**Figure 1:**
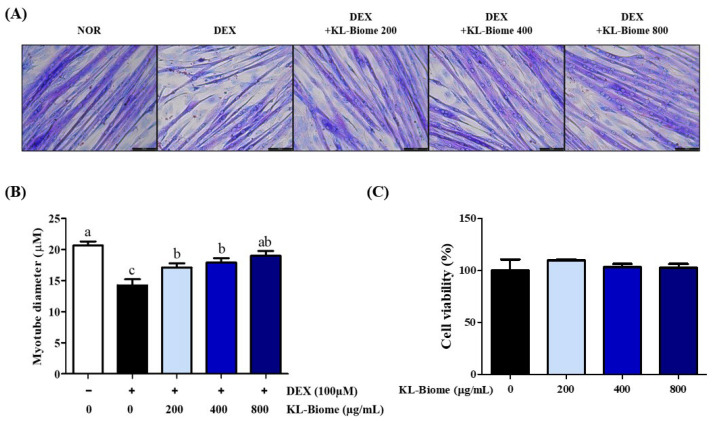
Effect of KL-Biome on myotube diameter in DEX-treated C2C12 myotubes. (**A**) Representative images of Jenner–Giemsa staining (scale bar: 100 μm), (**B**) myotube diameter, and (**C**) cell viability. The cells were incubated with KL-Biome in the presence or absence of DEX (100 μM) for 48 h. Data are expressed as the mean ± SEM. Different letters indicate significant differences at *p* < 0.05.

The effect of KL-Biome treatment on the diameter of DEX-induced C2C12 myotubes was examined using Jenner–Giemsa staining (Figure 1A). DEX treatment significantly decreased myotube diameter, suggesting the induction of muscle atrophy (Figure 1B, *p* < 0.05). In contrast, KL-Biome treatment significantly increased myotube diameter and was comparable to the normal control at a concentration of 800 µg/mL. This indicates that KL-Biome provides protection against DEX-induced muscle atrophy without causing cytotoxicity (Figure 1C).

As myoblasts differentiate into myotubes, myotube diameter increases. Conversely, glucocorticoids, such as DEX, lead to muscle loss by suppressing insulin-mediated glucose uptake and its utilization in skeletal muscles [17]. DEX is widely used to induce muscle atrophy in both cell cultures and animal models. DEX-induced muscle atrophy is considered an alternative model to naturally aged sarcopenia based on similarities in body composition and muscle strength change; nevertheless, certain discrepancies have been noted in the detailed muscle protein expression between DEX- and age-mediated muscle atrophy [18].

### 2.2. Effect of KL-Biome on Protein Degradation-Associated Gene Expression in DEX-Treated C2C12 Myotubes

**Figure 2:**
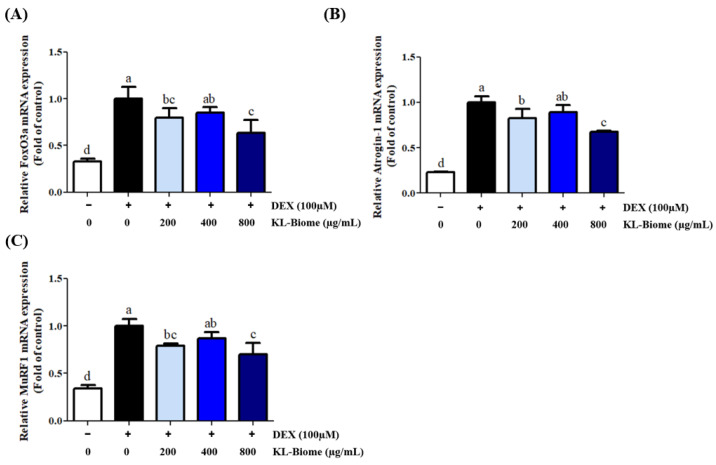
Effect of KL-Biome on protein degradation-associated gene expression in DEX-treated C2C12 myotubes. (**A**) FoxO3a, (**B**) Atrogin-1, and (**C**) MuRF1 gene expression. C2C12 myotubes were treated with KL-Biome in the presence or absence of 100 μM DEX for 24 h. Gene expression levels were analyzed using q-RT PCR, and normalization was performed based on the expression level of the housekeeping gene, GAPDH. Data are expressed as the mean ± SEM. Different letters indicate significant differences at *p <* 0.05.

To assess the effect of KL-Biome on protein degradation-associated gene expression, FoxO3a, atrogin-1, and MuRF1 levels were analyzed using qRT-PCR. The DEX-induced elevation of FoxO3a, atrogin-1, and MuRF1 gene expression levels was significantly downregulated by KL-Biome treatment (Figure 2, *p* < 0.05).

At a concentration of 800 µg/mL, the gene expression levels of FoxO3a, atrogin-1, and MuRF1 decreased by 36%, 33%, and 30%, respectively. However, exclusive treatment with the excipient (soybean powder) did not affect protein degradation-associated gene expression in DEX-treated C2C12 myotubes (Supplementary Figure S1).

Ubiquitination is a process targeting proteins for degradation that is mediated by ubiquitin ligases. The expression of E3 ubiquitin ligases, such as atrogin-1 and MuRF1, is typically upregulated in DEX-treated C2C12 myotubes, and ubiquitin ligases are activated by FoxO transcription factors [19]. MuRF1 is known to ubiquitinate major structural muscle proteins, such as myosin heavy chain and actin [20,21]. Atrogin-1 inhibits myogenesis by counteracting the expression of myogenic factors, such as MyoD, and suppresses the replacement of degraded muscle proteins [22]. The ubiquitin–proteasome system plays an important role in muscle homeostasis by selectively targeting protein degradation, and it is essential for muscle structure and function.

KL-Biome treatment effectively inhibited the expression of FoxO3a but subsequently downregulated that of atrogin-1 and MuRF1, suggesting that KL-Biome improves DEX-induced muscle atrophy by suppressing the protein degradation pathway. Consistent with the present study’s findings, *Lactobacillus rhamnosus* JY02-conditioned medium lowered atrogin-1 and MuRF1 expression levels in DEX-treated C2C12 myotubes [23].

### 2.3. Effects of KL-Biome on the Protein Expression of Akt, mTOR, and FoxO3a in DEX-Treated C2C12 Myotubes

**Figure 3:**
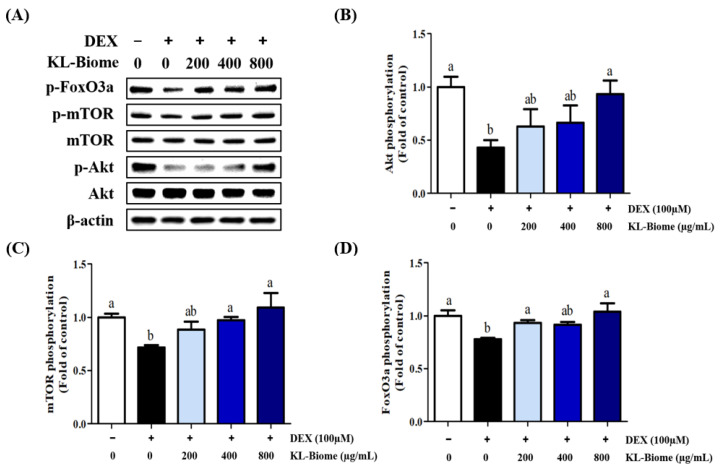
Effect of KL-Biome on Akt, mTOR, and FoxO3a expression in DEX-treated C2C12 myotubes. (**A**) Western blot image, and (**B**) Akt, (**C**) mTOR, and (**D**) FoxO3a protein expression. C2C12 myotubes were treated with KL-Biome in the presence or absence of 100 μM DEX for 48 h. Data are expressed as the mean ± SEM. Different letters indicate significant differences at *p <* 0.05.

To examine the effect of KL-Biome treatment on muscle protein synthesis, changes in the expression levels of Akt, mTOR, and FoxO3a in DEX-induced C2C12 cells were analyzed using western blotting (Figure 3). Akt, mTOR, and FoxO3a phosphorylation was significantly decreased by DEX treatment but significantly increased by KL-Biome treatment (*p <* 0.05). This indicates that KL-Biome potentially improves muscle synthesis via the Akt/mTOR/FoxO3a signaling pathway in muscle atrophy.

Akt is a key intermediate of the protein anabolic cascade and it modulates muscle protein mass via mTORC1 and FoxO1 signaling. mTOR, a serine/threonine kinase, stimulates protein synthesis by phosphorylating p70S6 Kinase 1 and eukaryotic initiation factor 4E-Binding Protein [24]. mTORC1 inhibition promotes ubiquitination in cells, thus increasing protein breakdown by the ubiquitin–proteasome system [25]. Akt-deficient skeletal muscle leads to mitochondrial dysfunction and damage to mitochondrial integrity, reflecting a shift toward non-oxidative metabolism [26]. In addition, Akt phosphorylation prevents the nuclear entry of FoxO3a and mitigates protein degradation by inhibiting atrogin-1 and MuRF1 [19]. Although some studies have indicated that mTORC1 signaling potentially elicits different consequences depending on the type of muscle and muscle atrophy, the Akt-dependent pathway serves a crucial role in maintaining muscle mass in muscle atrophy [27].

The lack of clear dose dependency, as shown in Figure 2 and Figure 3, might have emanated from the sample’s multiple nature. KL-Biome’s constituent heat-killed microbes and their metabolites are probably involved in multiple-target signaling pathways, leading to complex cellular responses. However, KL-Biome treatment consistently decreased protein degradation-associated gene expression but increased the phosphorylation of signaling proteins responsible for muscle synthesis.

### 2.4. Effects of KL-Biome on Body Composition and Muscle Weight in DEX-Treated Mice

**Figure 4:**
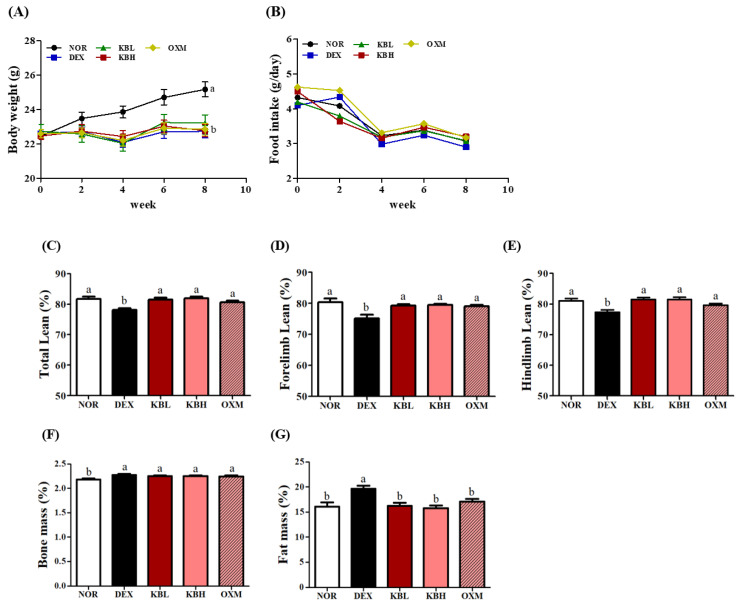
Effect of KL-Biome on body composition of DEX-treated mice. (**A**) Body weight, (**B**) food intake, (**C**) total lean mass, (**D**) forelimb lean mass, (**E**) hindlimb lean mass, (**F**) bone mass, and (**G**) fat mass. KBL: 900 mg KL-Biome/kg BW; KBH: 1800 mg KL-Biome/kg BW; OXM: 50 mg oxymetholone/kg BW. The data indicate mean ± SEM. Different letters indicate significant differences at *p <* 0.05 (n = 8).

The effect of KL-Biome on muscle atrophy in DEX-treated mice was evaluated after 8-week treatment with KL-Biome (900 and 1800 mg/kg). Body weight significantly decreased in all DEX-treated groups compared with that in the non-treated control (NOR) group (Figure 4A). No significant differences in food intake were observed among the groups (Figure 4B). According to dual-energy X-ray absorptiometry (DEXA), the DEX group exhibited a significant reduction in total lean mass compared with the NOR group (*p <* 0.05).

The low- and high-dosage KL-Biome groups (KBL and KBH, respectively) and the positive control—the oxymetholone group (OXM)—exhibited significant increases in total lean mass compared to the DEX group (Figure 4C, *p <* 0.05). No difference in total lean mass was observed between the KBL and KBH groups. The lean masses of the forelimbs and hindlimbs displayed the same trend as that observed in total lean mass (Figure 4D,E). While bone mass showed no significant differences, fat mass displayed significant differences between the DEX and other groups (Figure 4F,G).

In accordance with the report of Furukawa et al. [28], DEX-treated groups resulted in weight reduction compared with the NOR group. The detailed molecular mechanisms underlying DEX-mediated decreases in lean mass can vary depending on the experimental factors, such as dosage, duration, and point of determination. Myostatin is involved in the early stages of DEX treatment (5 days), while E3 ligases, such as MuRF-1 and atrogin, are more closely linked to the later stages (>10 days) of the atrophic response [29].

**Figure 5:**
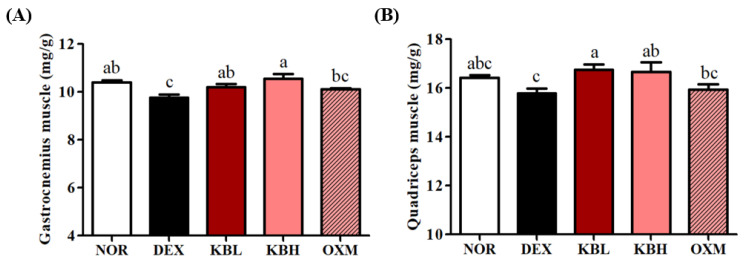
Effect of KL-Biome on muscle mass in DEX-treated mice. (**A**) Gastro muscle mass and (**B**) quad muscle mass. Muscle mass was normalized to the total body weight of individual mice. KBL: 900 mg KL-Biome/kg BW; KBH: 1800 mg KL-Biome/kg BW; OXM: 50 mg oxymetholone/kg BW. Data are expressed as the mean ± SEM. Different letters indicate significant differences at *p <* 0.05 (n = 8).

Changes in mouse hindlimb muscle mass were confirmed immediately after sacrifice. The weights of the gastro and quad muscles were normalized to the total body weight of the individual mice. These weights significantly decreased in the DEX group compared with those of the NOR group (*p <* 0.05); however, the KL-Biome groups (KBL and KBH) showed significant increases in muscle mass, as indicated by the DEXA measurements (Figure 5A,B, *p <* 0.05).

DEX-induced muscle atrophy reduced the number of fast-twitch fibers, suggesting a transition from fast- to slow-twitch muscle types [30]. The quad muscle chiefly comprises fast-twitch fibers, while the gastro muscle is a mixture of fast- and slow-twitch fibers [31]. These results indicate that the DEX-mediated decrease in mouse muscle mass was effectively attenuated by KL-Biome administration.

Again, no significant difference in muscle mass existed between the KBL and KBH groups suggesting that increasing KL-Biome dosage does not further improve DEX-induced muscle atrophy. Most previous animal studies on the improvement of muscle atrophy using lactic acid bacteria predominantly focused on their probiotic function [14,15], and considerably limited information was available to determine the appropriate postbiotic dosage. Therefore, in the present study, KBL was generously administered at sufficient levels to significantly improve muscle atrophy.

### 2.5. Effect of KL-Biome on Biochemical Markers in DEX-Treated Mice

KL-Biome treatment-induced changes in serum biochemical markers were analyzed in the DEX-treated mice (Table 1). Creatine phosphokinase (CPK) levels significantly increased in the DEX group (*p <* 0.05) but significantly decreased upon KL-Biome treatment (KBL and KBH) (*p <* 0.05). KL-Biome also significantly lowered lactate dehydrogenase (LDH) levels (*p <* 0.05). CPK and LDH are muscular enzymes involved in energy metabolism and they serve as serum biochemical markers, reflecting muscle cell damage [32]. These enzymes potentially leaked into the serum upon muscle damage, and their serum levels significantly decreased in mice with improving muscle atrophy, as evidenced by the present study [33].

Serum ALT, AST, and BUN levels were analyzed to evaluate the potential toxicity of KL-Biome. AST levels in the DEX-treated groups significantly increased compared with those of the NOR group (*p <* 0.05), but no differences in serum ALT and AST levels were noted among the DEX-treated groups. Since AST participates in hepatic gluconeogenesis, chronic DEXA treatment may increase serum AST levels by increasing gluconeogenesis [34]. BUN, a renal toxicity index, did not yield any significant differences among the treatment groups. These results indicate that KL-Biome administration does not cause hepatic or renal toxicity in mice and may offer partial protection against DEX-induced muscle damage.

### 2.6. Effects of KL-Biome on Muscle Function in DEX-Treated Mice

**Figure 6:**
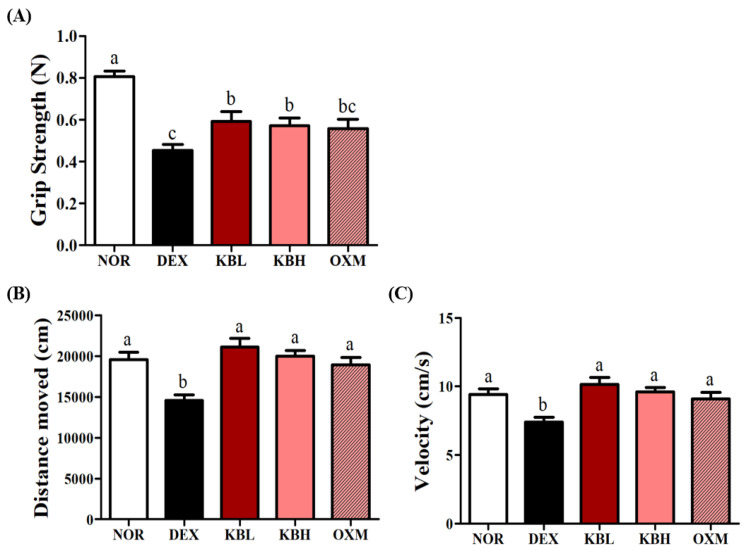
Effect of KL-Biome on muscle function in DEX-treated mice. (**A**) Grip strength, (**B**) moving distance, and (**C**) velocity. KBL: 900 mg KL-Biome/kg BW; KBH: 1800 mg KL-Biome/kg BW; OXM: 50 mg oxymetholone/kg BW. Data are expressed as the mean ± SEM. Different letters indicate significant differences at *p <* 0.05 (n = 8: grip strength, n = 4: treadmill exercise capacity).

Muscle strength is known to decrease with muscle atrophy [35]. To ascertain whether KL-Biome administration ameliorates the DEX-induced decline in skeletal muscle function, grip strength and exercise performance tests were conducted. DEX treatment significantly decreased grip strength. Nonetheless, KL-Biome administration significantly improved grip strength reflecting the restoration of muscular strength (*p <* 0.05, Figure 6A).

Treadmill exercise capacity was used to evaluate muscle function. The DEX group displayed the lowest values for both moving distance and speed in the treadmill exercise test. In contrast, the KL-Biome groups recovered moving distance and speed comparable to the NOR group (Figure 6B,C, *p <* 0.05). Muscle fiber type can change from fast- to slow-twitch upon prolonged DEX exposure [36]. This change attenuates muscle strength and increases sensitivity to fatigue [37]. These findings suggest that KL-Biome administration effectively suppresses DEX-induced muscle function impairment.

### 2.7. Effect of KL-Biome on Structural Damage of Muscle Tissues in DEX-Treated Mice

**Figure 7:**
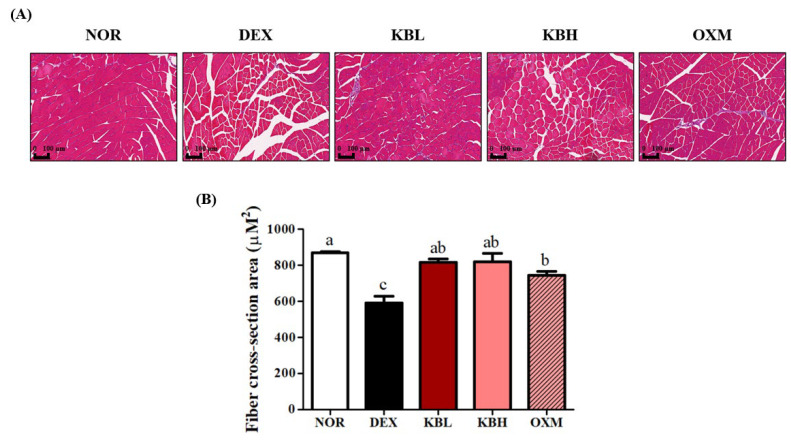
Effect of KL-Biome on structural damage to gastrocnemius muscle tissue in DEX-treated mice. (**A**) H&E staining of gastro muscle and (**B**) muscle fiber CSA. KBL: 900 mg KL-Biome/kg BW; KBH: 1800 mg KL-Biome/kg BW; OXM: 50 mg oxymetholone/kg BW. Data are expressed as the mean ± SEM. Different letters indicate significant differences at *p <* 0.05 (n = 5).

A common characteristic of skeletal muscle atrophy is decreased muscle fiber size, which can be assessed by quantifying the fiber cross-sectional area (CSA) of a fixed sample [38]. To examine the extent of muscle atrophy caused by DEX or DEX+ KL-Biome treatment, the gastro muscle was stained with H&E and CSA was calculated. The DEX group exhibited the greatest reduction in muscle fiber size, and average muscle fiber CSA decreased by 65%. Moreover, large inter-fiber gaps were observed (Figure 7A). KL-Biome administration alleviated DEX-induced structural damage resulting in significant CSA recovery comparable to that of the NOR group (Figure 7B). *L. rhamnosus* JY02 administration improved muscle fiber CSA in DEX-induced atrophy in mice; however, the authors failed to find significant differences in quadricep and gastrocnemius muscle weight [23]. Based on lean muscle mass, fiber CSA, grip strength, and the treadmill test results, KL-Biome administration effectively prevented muscle atrophy and dysfunction in the DEX group.

### 2.8. Effect of KL-Biome on Major Signaling Factors Affecting Protein Synthesis and Degradation in DEX-Treated Mice

Skeletal muscle homeostasis is maintained through a delicate balance between protein synthesis and breakdown [39]. The effects of KL-Biome treatment on the expression of major signaling factors in the gastrocnemius muscles of DEX-treated mice were analyzed. Only KBL (800 mg/kg BW) was used for western blotting because no significant difference existed between KBL and KBH in the phenotypic measurements, such as muscle mass, grip strength, exercise capability, and CSA.

**Figure 8:**
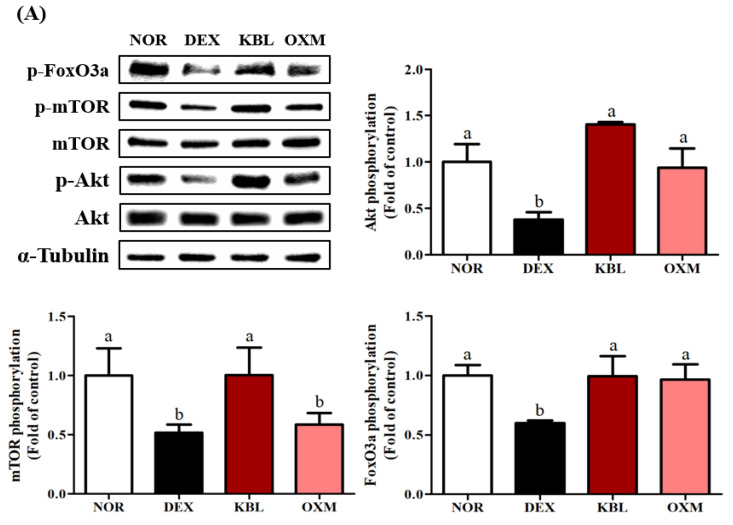

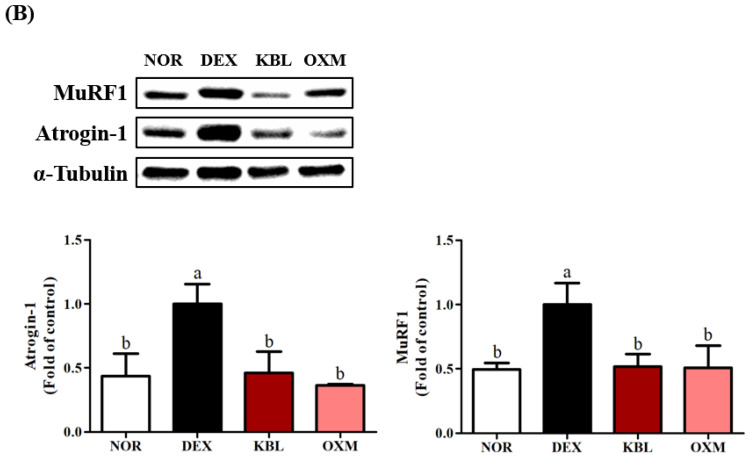
Effect of KL-Biome on major signaling factors affecting protein synthesis and degradation in DEX-treated mice. (**A**) Protein synthesis-related protein expression and (**B**) protein degradation-related protein expression. KBL: 900 mg KL-Biome/kg BW; OXM: 50 mg oxymetholone/kg BW. Data are expressed as the mean ± SEM. Different letters indicate significant differences at *p <* 0.05 (n = 3).

KBL administration consistently stimulated Akt, mTOR, and FoxO3a phosphorylation, suggesting the restoration of muscle protein synthesis (Figure 8A, *p <* 0.05). In terms of catabolic signaling, KL-Biome treatment significantly downregulated the DEX-mediated elevated expression of Arogin-1 and MuRF1 (Figure 8B, *p <* 0.05).

The Akt/mTOR pathway is a key regulator of skeletal muscle hypertrophy, and its activation preserves muscle fiber size and prevents muscle atrophy [40]. In turn, Akt phosphorylation suppresses the nuclear translocation of FoxO3a, which coordinately activates the ubiquitin–proteasome pathway and involves MuRF-1 and atrogin-1 [25]. These results are consistent with those of in vitro assays using C2C12 myotubes. Taken together, KL-Biome administration effectively improved DEX-induced muscle atrophy by modulating the expression of both muscle protein synthesis and degradation.

### 2.9. Effect of KL-Biome on Gut Microbiome Composition

**Figure 9:**
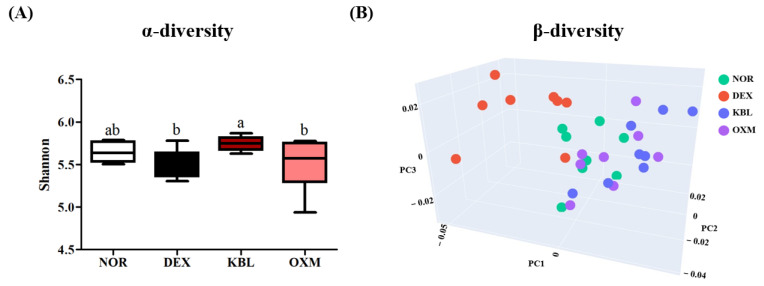
Effect of KL-Biome on gut microbiota diversity. (**A**) α-diversity and (**B**) β-diversity. KBL: 900 mg KL-Biome/kg BW; OXM: 50 mg oxymetholone/kg BW. Data are expressed as the mean ± SEM. Different letters indicate significant differences at *p <* 0.05 (n = 8).

Aging causes an imbalance in the gut microbiome leading to a reduction in microbial diversity and beneficial bacterial metabolites [41]. Whole-genome shotgun sequencing analysis was performed to examine changes in gut microbial diversity and composition. The KBL group displayed greater α-diversity than the DEX group (Figure 9A, *p <* 0.05).

β-diversity analysis revealed that KBL and OXM group clusters were clearly differentiated from the DEX group and had similar distribution patterns to those of the NOR group (Figure 9B).

**Figure 10:**
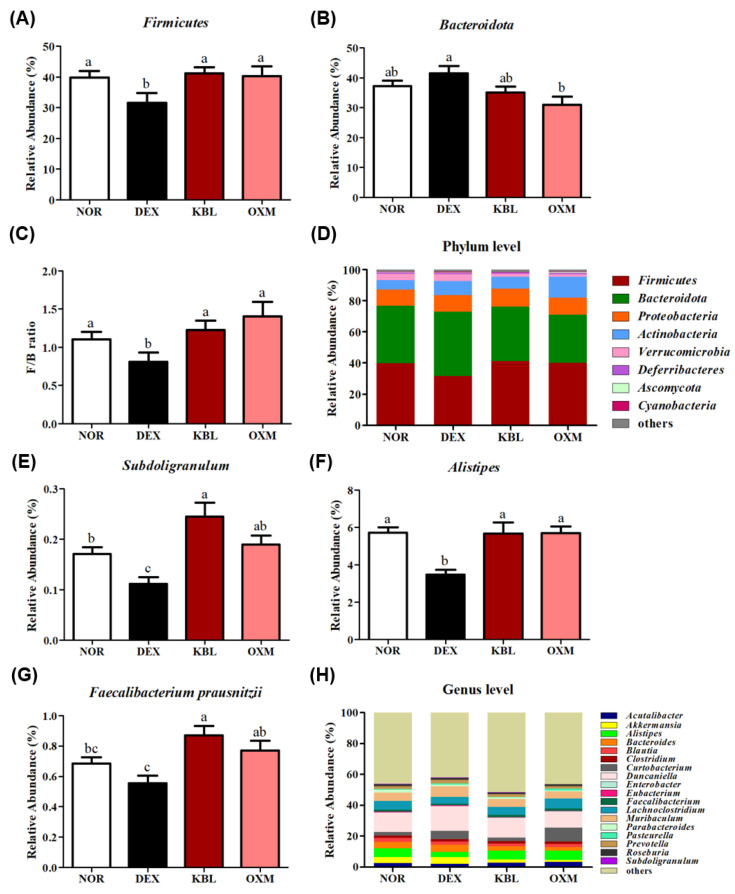
Effect of KL-Biome on the cecal microbiota. (**A**) Relative abundance of *Firmicutes*, (**B**) relative abundance of *Bacteriodetes*, (**C**) *Firmicutes*/*Bacteroidetes* ratio, (**D**) taxonomic analysis at the phylum level, (**E**) relative abundance of *Subdoligranulum*, (**F**) relative abundances of *Alistipes*, (**G**) *Faecalibacterium prausnitzii*, and (**H**) taxonomic analysis at the genus level. KBL: 900 mg KL-Biome/kg BW; OXM: 50 mg oxymetholone/kg BW. Data are expressed as the mean ± SEM. Different letters indicate significant differences at *p <* 0.05 (n = 8).

α-diversity is regarded as an indicator of gut health and reflects the host’s disease status, while β-diversity indicates the stability and ecological function of the gut microbiota [42]. Qiu et al. [43] found that DEX treatment significantly decreased α-diversity and the *Firmicutes*/*Bacteroidetes* (F/B) ratio. The F/B ratio was also significantly reduced in patients with chronic liver disease with low muscle mass [44]. The F/B ratio decreased in the DEX group but significantly increased after KL-Biome administration (Figure 10A–C). These results suggest that KL-Biome administration helps maintain gut microbial diversity and reverts DEX-induced dysbiosis closer to a normal healthy state.

SCFA production is positively associated with skeletal muscle mass. Frampton et al. [45] reported that SCFAs, especially butyrate, act as potential regulators of skeletal muscle mass and function during the aging process. Butyrate treatment improved mitochondrial biogenesis in skeletal muscle but decreased intermuscular fat accumulation in old mice [46]. At the genus level, the relative abundances of *Subdologranulum* and *Alistipes* significantly decreased in the DEX group. In contrast, they significantly increased following KL-Biome administration (Figure 10E–G).

*Subdoligranulum* expresses acetyl-CoA acetyltransferase and butyrate kinase, which are involved in the butyrate-production pathway [47]. Fecal butyrate levels significantly decreased in older adults with low muscle mass, and a positive correlation was established between butyrate and *Subdoligranulum* [48]. The genus *Alistipes* is also considered a potential SCFA producer. The relative abundance of *Alistipes* increased with increasing healthy phenotypes of various diseases, such as colitis and hepatic fibrosis [49]. *Faecalibacterium prausnitzii* is a well-known SCFA producer that abundantly populates the gut microbiota of healthy individuals; it is a promising probiotic for the alleviation of gut dysbiosis and inflammatory bowel disease [50].

The oral administration of *Faecalibacterium prausnitzii* EB-FPDK11 (10^8^ CFU/day) for 5 weeks significantly improved grip strength and inhibited the gene expression of skeletal muscle degradation in an immobilization-induced muscle atrophy mouse model [51]. Lv et al. [52] demonstrated that gut microbial butyrate synthesis was significantly associated with the skeletal muscle index in healthy menopausal women, and *Faecalibacterium prausnitzii* and *Butyricimonas virosa* were strongly related to increased butyrate synthesis.

Taken together, KL-Biome administration stimulated butyrate-producing bacteria, such as *Subdologranulum*, *Alistipes*, and *Faecalibacterium prausnitzii.* The increased SCFA bioavailability elicited by KL-Biome administration potentially contributes to muscle function by providing energy via direct oxidation or by promoting fatty acid oxidation. SCFAs can improve anabolic resistance in skeletal muscles by enhancing insulin sensitivity, mitochondrial biogenesis, and inflammation [8]. Prolonged exposure to DEX significantly decreased gut microbiota diversity in rats and increased the abundance of proteobacteria, reflecting a proinflammatory state [53]. In this regard, mitigating inflammation via KL-Biome treatment may help inhibit the catabolic response in skeletal muscles. Therefore, KL-Biome administration exerts beneficial effects on muscle mass and function by increasing the abundance of specific microbes, such as SCFA producers.

## 3. Materials and Methods

### 3.1. Materials

C2C12 myoblasts were purchased from the American Type Culture Collection (Manassas, VA, USA). Dulbecco’s modified Eagle medium (DMEM), penicillin-streptomycin (P/S), and fetal bovine serum (FBS) were procured from Welgene (Daegu, Republic of Korea). Horse serum (HS) was acquired from Gibco (Grand Island, NY, USA). Dexamethasone (DEX) and other chemicals were obtained from Sigma-Aldrich Chemical Co., Ltd. (St. Louis, MO, USA). The High-Capacity RNA-to-cDNA Kit, TaqMan^®^ Gene Expression Master Mix, and TaqMan^®^ probes (5′-fluorescein based reporter dye; 3′-TAMRA™ quencher) were secured from Applied Biosystems (Foster City, CA, USA). All antibodies used for western blotting were obtained from Cell Signaling Technology (Danvers, MA, USA).

### 3.2. Preparation of KL-Biome

*L. plantarum* KM2 was inoculated (2%, *v*/*v*) into lactic acid bacteria production medium and incubated at 30 °C for 12 h. Thereafter, the *L. plantarum* KM2 was heated at 90 °C for 1 h and the cell-free supernatant (CFS-*L. plantarum*) was collected using a centrifuge followed by filtration through a 0.2 μm membrane. The CFS-*L. plantarum* was subsequently freeze-dried with an excipient (7%, soybean powder, *w*/*v*). The dried centrifugal precipitate containing heat-killed microbials was mixed with CFS powder. The formulated postbiotic ingredient comprising heat-killed *L. plantarum* KM-2 bacteria and their metabolites as well as the excipient was named “KL-Biome”.

### 3.3. C2C12 Myoblasts Cell Culture

C2C12 myoblasts were maintained in DMEM containing 10% FBS and 1% P/S at 37 °C under 5% CO_2_ conditions. When the myoblasts had reached 100% confluence, their differentiation into myotubes was induced with DMEM containing 2% HS. After 6 days, muscle atrophy was induced via DEX (100 µM) addition in the presence or absence of KL-Biome (200, 400, and 800 µg/mL) for 24 h. Cytotoxicity of KL-Biome was evaluated using the 3-(4,5-dimethylthiazol-2-yl-2,5-diphenyltetrazolium bromide assay.

### 3.4. Myotube Diameter

Myotube diameter was measured from the images differentiated using Jenner–Giemsa-stained C2C12 cells [54]. Briefly, C2C12 cells were fixed with absolute methanol and treated with Jenner staining solution. After 10 min, the specimen was washed thrice with distilled water. The cells in each well were observed using a Leica DM IL LED microscope (Leica, Wetzlar, Germany). Representative images from three randomly selected fields were captured and myotube diameter was quantified using LAS X software (version 3.7.4; Leica Microsystems Ltd., Germany)

### 3.5. DEX-Induced Atrophy Mouse Model Microsystems Ltd.

Seven-week-old male C57BL/6J mice were obtained from Orient Bio Inc. (Seongnam, Republic of Korea) and acclimatized for 1 week at constant temperature (22 ± 2 °C) and humidity (50 ± 10%) under a 12 h light/dark cycle. The animal experiments were approved by the Kookmin University Institutional Animal Care and Use Committee (KMU-2023-05). After adaptation, the mice were randomly assigned to the following five groups (n = 8, 4 mice/cage): the normal group (NOR), DEX-treated control (DEX), low-dose (900 mg KL-Biome/kg BW) KL-Biome (KBL), high-dose KL-Biome (1800 mg KL-Biome/kg BW) (KBH), and oxymetholone (50 mg/kg BW) (OXM; positive control) groups. KL-Biome and OXM were administered orally via gavage daily for 8 weeks, while an equal volume of saline was administered to the NOR and DEX groups. DEX (5 mg/kg) was intraperitoneally administered to all mice except the NOR group mice. All mice had free access to a chow diet (5L79, Orient Bio Inc., Seongnam, Republic of Korea) and water. Food intake and body weight were measured every 2 weeks.

### 3.6. Body Composition

Body composition including lean, fat, and bone masses was measured using dual-energy X-ray absorptiometry (DEXA; Medikors Inc., Seongnam, Republic of Korea). Mouse forelimb and hindlimb specimens were selected using a region of interest. The lean mass of each left and right limb was measured, and the average values were determined.

### 3.7. Blood and Tissue Collection and Serum Biochemical Analysis

Whole blood, gastrocnemius (gastro) muscle tissue, and quadriceps (quad) muscle tissue were collected on the day of euthanasia. Serum was immediately separated by centrifugation (3000× *g*, 10 min, 4 °C). Gastro and quad muscles were immediately weighed and rapidly frozen in a −80 °C freezer. Serum creatine phosphokinase (CPK), lactate dehydrogenase (LDH), aspartate aminotransferase (AST), alanine aminotransferase (ALT), and blood urea nitrogen (BUN) were analyzed using a chemical analyzer (Fujifilm Dri-Chem 3500i, Fuji Photo Film, Ltd., Tokyo, Japan).

### 3.8. Grip Strength Test

Forelimb grip strength was determined after muscle atrophy induction using a grip strength meter (DBI, Co., Eumsung, Republic of Korea) equipped with a T-bar. Each mouse was placed on the bar and its tail slowly pulled back until the grip was released. Grip strength was measured three times for each mouse.

### 3.9. Exercise Performance Test

The mouse running test was performed on a treadmill machine. Prior to the exercise performance test, a 2-day training session was conducted at a speed of 10 m/min and a 10° incline for 10 min. After warming up, the speed was gradually increased by 2 m/min every 3 min until a maximum speed of 30 m/min was achieved and maintained. The entire running process was recorded and the video lengths for all groups were standardized to ensure consistent behavioral analysis. Moving distance and speed were analyzed using EthoVision^®^ XT video tracking software (version 17.5; Noldus Information Technology, Wageningen, The Netherlands).

### 3.10. Histological Analysis

Gastro muscle tissues were fixed in 4% formaldehyde and subsequently embedded in paraffin. Thereafter, they were sectioned into 4 µM specimens and stained with hematoxylin and eosin (H&E). Muscle fiber images (20× magnification) were captured using KFBIO Slide Manager (KFBIO, Ningbo, China) and fiber CSA (µm^2^) was measured using ImageJ software (version 1.8.0; National Institutes of Health, Bethesda, MD, USA). The average CSA was calculated from 100 fibers per mouse.

### 3.11. RNA Extraction and Quantitative Real-Time Polymerize Chain Reaction (qRT-PCR)

Total RNA was isolated from C2C12 cells using NucleoZOL reagent (Macherey-Nagel, Düren, Germany) and gastro muscle using TRIzol reagent (Invitrogen, Carlsbad, CA, USA). Complementary DNA (cDNA) was synthesized using the High-Capacity RNA-to-cDNA Kit (Applied Biosystems). The cDNA was mixed with TaqMan^®^ Gene Expression Master Mix (Applied Biosystems) and qRT-PCR was performed using the StepOne Plus^TM^ RT-PCR System (Applied Biosystems). The following primers were used in the analysis: GAPDH (Mm99999915_g1), FoxO3a (Mm01185722_m1), atrogin-1 (Mm00499523_m1), and MuRF1 (Mm01185221_m1). Target gene expression was normalized using GAPDH (Mm99999915_g1).

### 3.12. Western Blot Analysis

Total lysates of C2C12 cells or gastro muscle tissue were homogenized in radioimmunoprecipitation assay lysis and extraction buffer (Thermo Fisher Scientific, Rockford, IL, USA) containing 1% protease inhibitor and 1% phosphate inhibitor. Immunoblotting was performed as previously described using the following primary antibodies [55]: β-actin (Cat. 4970S, Cell Signaling Technology), AKT (Cat. 4691S), p-AKT (Cat. 4060S), mTOR (Cat. 2986S), p-mTOR (Cat. 5536S), and p-FoxO3a (Cat. 9466S).

### 3.13. Cecal Gut Microbiota Analysis via Shotgun Sequencing

Mouse cecal DNA was extracted using the QIAamp Fast DNA Stool Mini Kit (Qiagen, Valencia, CA, USA) and DNA concentration was quantified using a Qubit^®^4.0 Fluorometer (Invitrogen, U.S.). Metagenomic sequencing libraries were constructed with ≥150 ng of isolated DNA using the Illumina TruSeq Nano DNA Library Kit (Illumina, Inc., San Diego, CA, USA). Paired-end metagenomic sequencing (150-bp PE reads) was conducted using the Illumina NextSeq2000 Sequencing System. Low-quality bases and adapter artifacts in raw sequencing were eliminated from shotgun metagenomic analysis using Trimmomatic v0.39. Shotgun taxonomic classification was performed using Kraken v2.1.2 (a k-mer matching algorithm). After taxonomic classification, species-to-phylum bacterial abundance was refined using Bracken [56]. KL-Biome-induced changes in microbial community composition were characterized using rarefaction analysis and Shannon diversity calculations (unweighted UniFrac metrics).

### 3.14. Statistical Analysis

Data are presented as the mean ± standard error of the mean (SEM). Statistical analyses were performed using SPSS 28.0 software (Chicago, IL, USA). One-way analysis of variance was used to compare data, and Duncan’s multiple-range comparison test was applied to determine significant differences (*p <* 0.05) among treatment means.

## 4. Conclusions

KL-Biome treatment effectively restored muscle atrophy by downregulating the catabolic ubiquitin–proteasome pathway and upregulating the anabolic Akt/mTOR/FoxO3a pathway. Furthermore, it significantly and consistently improved various biomarkers representing muscle mass, strength, function, and damage in DEX-treated atrophy mice. The gut microbiota composition of the KL-Biome group exhibited a similar change trend to that of the NOR group, displaying an increase in the relative abundance of SCFA producers. Based on these findings, KL-Biome exerts beneficial effects against muscle atrophy and is potentially useful as a postbiotic supplement capable of enhancing muscle strength and function.

## Figures and Tables

**Table 1:** Effect of KL-Biome on serum muscle damage and toxicity markers in DEX-treated mice.

Group	CPK (U/L)	LDH (U/L)	ALT (U/L)	AST (U/L)	BUN (mg/dL)
NOR	187.00 ± 62.34 ^b^	288.50 ± 73.19 ^a^	25.25 ± 6.88 ^c^	38.88 ± 9.31 ^b^	24.60 ± 2.76 ^a^
DEX	340.88 ± 154.51 ^a^	295.63 ± 65.91 ^a^	31.13 ± 2.30 ^bc^	59.25 ± 10.07 ^a^	24.15 ± 1.57 ^a^
KBL	147.50 ± 101.33 ^b^	99.25 ± 15.74 ^c^	29.00 ± 5.35 ^bc^	52.88 ± 10.22 ^a^	21.39 ± 1.13 ^b^
KBH	199.63 ± 56.45 ^b^	137.00 ± 18.59 ^c^	32.00 ± 3.42 ^b^	62.75 ± 12.28 ^a^	22.90 ± 1.09 ^ab^
OXM	99.13 ± 35.13 ^b^	211.38 ± 46.99 ^b^	43.75 ± 7.96 ^a^	58.88 ± 11.73 ^a^	24.74 ± 1.47 ^a^

CPK, creatine phosphokinase; LDH, lactate dehydrogenase; ALT, alanine aminotransferase; AST, aspartate aminotransferase; BUN, blood urea nitrogen. KBL: 900 mg KL-Biome/kg BW; KBH: 1800 mg KL-Biome/kg BW; OXM: 50 mg oxymetholone/kg BW. Data are expressed as the mean ± SEM. Different letters indicate significant differences at *p <* 0.05 (n = 8).

## Data Availability

Data used in this study are available from the corresponding author J.I. upon reasonable request.

## Supplementary Materials

The following supporting information can be downloaded at: https://www.mdpi.com/article/10.3390/ijms25137499/s1.
