# Supplementary material for: KL-Biome (Postbiotic Formulation of Lactiplantibacillus plantarum KM2) Improves Dexamethasone-Induced Muscle Atrophy in Mice
Source: Int J Mol Sci. 2024 Jul 8;25(13):7499. doi: 10.3390/ijms25137499 (PMC11242066; doi:10.3390/ijms25137499)
Supplement: Supplementary file 1 [file ijms-25-07499-s001.zip › ijms-3094754-supplementary.pdf]

Supplement 1.

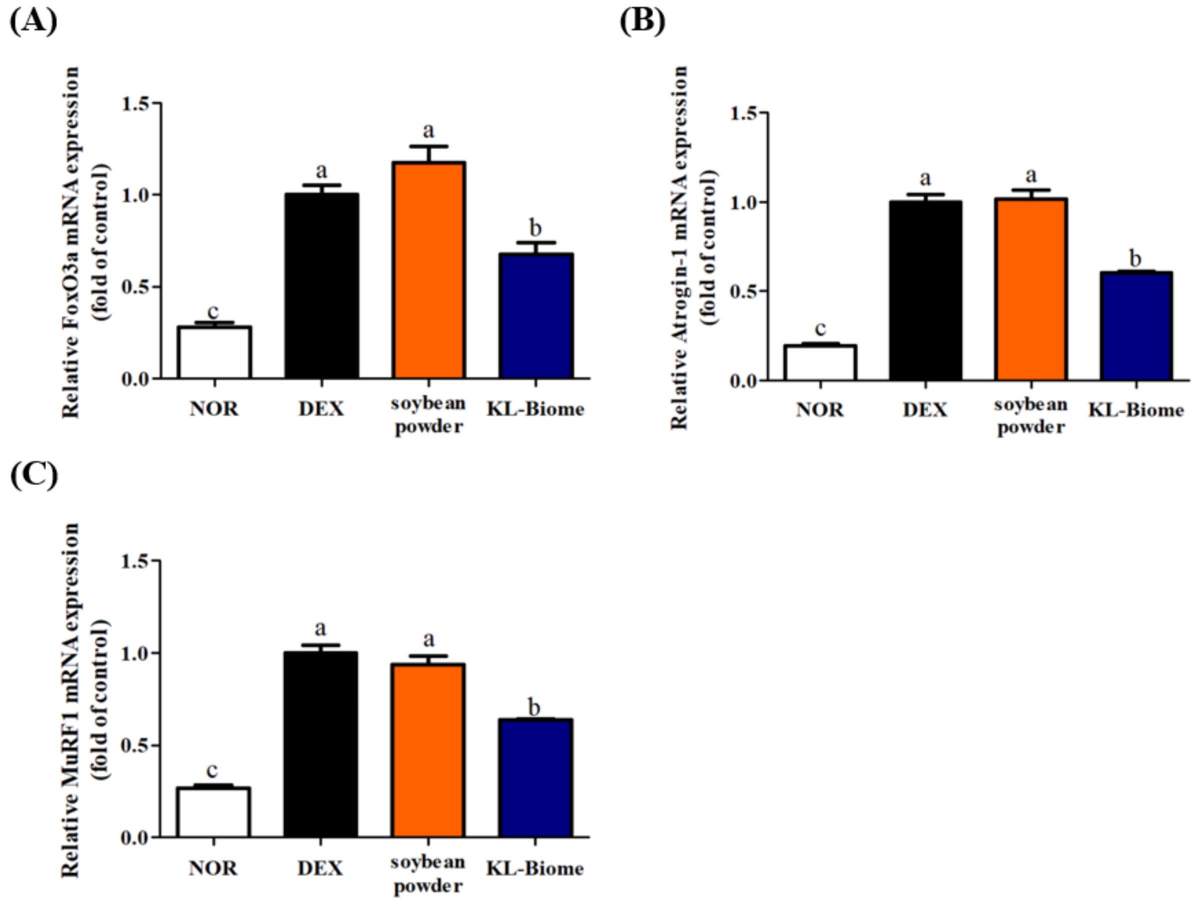

Figure S1. Effect of KL-Biome and incipient (soybean powder) on protein degradation-associated genes expressions in DEX-treated C2C12 myotubes. (A) FoxO3a, (B) Atrogin-1, and (C) MuRF1 genes expression. C2C12 myotubes were treated with incipient (soybean powder) and KL-Biome in the presence or absence of 100 μM DEX for 24 h. The samples had the equal concentration of incipient (400 ug/mL). The gene expression levels were analyzed using q-RT PCR, and normalization was performed based on the expression level of the housekeeping gene, GAPDH. The data are expressed as the mean ± SEM. Different letters indicate significant differences at $P<0.05$.

To assess the effect of CFS-*L. plantarum* KM2 culture and heat-treated microbe enhancing muscle strength, soybean powder (Sochengja) was employed as a carrier medium. Sochengja was administered at a concentration of 450 μg/mL, equivalent to that found in KL-Biome at 800 μg/mL. Subsequently, the expression levels of protein degradation-related factors were assessed using q-RT PCR.

Treatment with sochengja did not lead to a significant reduction in mRNA expression levels of FoxO3a, Atrogin-1, or MuRF1 (Fig. S1). However, treatment with KL-Biome containing CFS-*L. plantarum* KM2 culture and heat-treated microbe resulted in a significant decrease in expression levels.

These findings confirm that the reduced expression levels of protein degradation-related factors in DEX-induced muscle atrophy C2C12 myotubes is specifically attributable to the presence of CFS-*L. plantarum* KM2 culture and heat-treated microbe within the KL-Biome formulation.
